# Training Psychiatry with AI: Simulator with 100 Cases and Image-Based Multi Level Communication

**DOI:** 10.1192/j.eurpsy.2026.10627

**Published:** 2026-07-31

**Authors:** D. Novak, F. Bodnár, P. Kubíček, J. Vevera

**Affiliations:** 1 Department of Cybernetics, Czech Technical University in Prague, Prague; 2 Faculty of Medicine in Pilsen, Charles University, Pilsen, Czech Republic

## Abstract

**Introduction:**

We present a conversational-AI platform for simulation-based psychiatric training. The platform includes **100 clinically grounded use cases** across three communication-difficulty tiers (cooperative, resistant, and non-cooperative), enabling practice in adaptive questioning and de-escalation. During each interview, the system renders a **time-ordered series of simple avatar images.** When a medication is mentioned, students can open the integrated drug module to view the **package leaflet**.

**Objectives:**

We aim to extend our conversational-AI virtual-patient platform into a comprehensive, clinically curated set of **100 psychiatry use cases**, and to examine how **graded communication difficulty** shapes learners’ questioning strategies. To support presence and engagement, we added **simple avatar visuals** that depict only the patient’s physical appearance. When medications arise in dialogue, an integrated **drug module** reveals the **package leaflet** enabling context-aware medication education.

**Methods:**

Users interview a virtual patient via text or voice; a large language model (LLM) pipeline maps utterances to intents and generates guarded, diagnosis-consistent replies. The library now comprises **100** clinician-curated patient profiles derived from real cases, de-identified records, and carefully synthesised data, spanning diverse diagnostic scenarios (Fig. 1). During interviews, the system renders a simple avatar sequence to visualise the patient’s physical appearance (not mental-status change). Built-in tools let learners inspect relevant physiological/clinical examinations (Fig. 2), while an embedded assessment engine evaluates adherence to ethical and methodological standards and verifies diagnostic accuracy (Fig. 3).

**Results:**

Building on our prior pilot, where psychiatrists rated dialogue realism highly and students reported improved confidence with an average diagnostic score of ~71%, the expanded platform functioned reliably at scale with **100** curated cases. System latency remained low; trainees engaged smoothly with **three communication tiers**, and interaction logs showed expected shifts toward more targeted, clarifying questions in “so-so” and non-cooperative scenarios. The **avatar visuals** were well-received for anchoring patient identity without implying clinical state. When medications were mentioned, learners successfully used the integrated **drug module.**

**Image 1:**
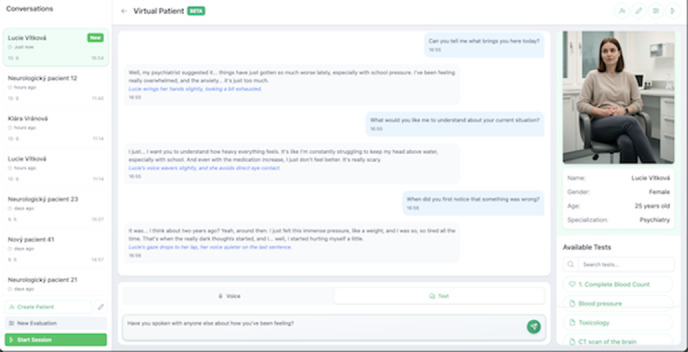
Image 1: Long description.

**Image 2:**
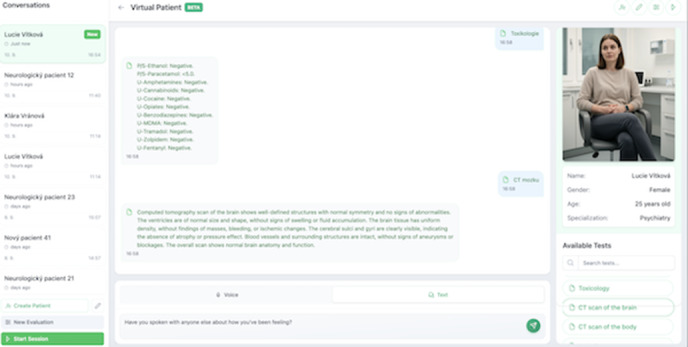
Image 2: Long description.

**Image 3:**
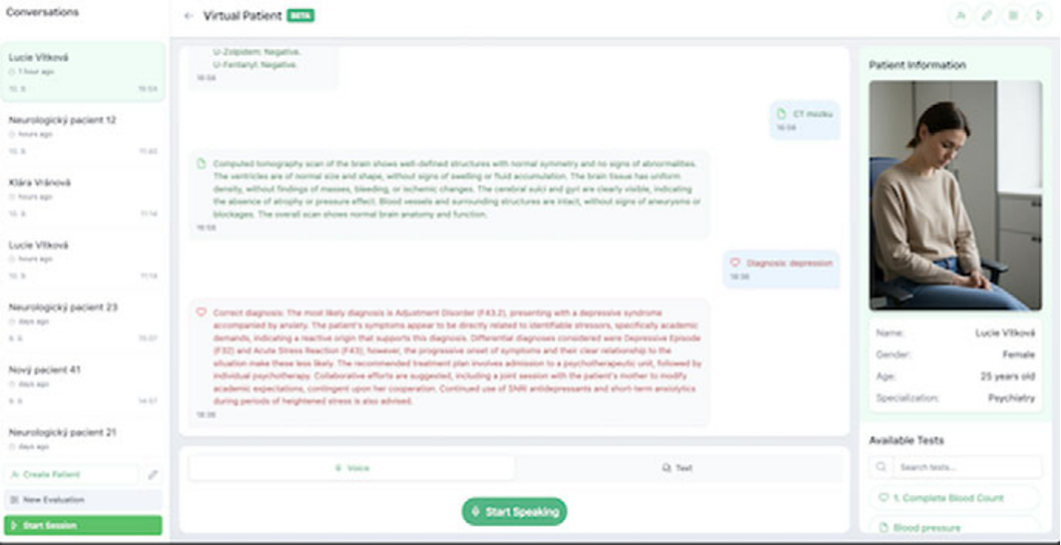
Image 3: Long description.

**Conclusions:**

A conversational-AI psychiatry simulator with graded communication difficulty, simple avatar visuals, and an in-context drug module is feasible and well-accepted, supporting medication-safety reasoning. Interaction analytics indicate more targeted questioning as difficulty increases, while learners efficiently retrieve drug information in context. The platform is ready for curriculum integration with automated feedback and educator dashboards; comparative outcomes will be reported.

**Disclosure of Interest:**

None Declared

## Long descriptions

### Image 1: Long description

The interface is divided into three main vertical sections.

1. Left Sidebar: Titled Conversations, it lists several patient records including Lucie Vitková marked as New, and various Neurologický pacient entries. At the bottom are buttons for Create Patient, New Evaluation, and a green Start Session button.

2. Central Chat Panel: Titled Virtual Patient BETA. It displays a dialogue between a clinician in blue bubbles and the patient in white bubbles. The patient, Lucie, describes feeling overwhelmed and anxious. Italicized blue text provides behavioral observations such as Lucie wrings her hands slightly and Lucie's gaze drops to her lap. Below the chat is a toggle for Voice or Text input and a text entry field asking Have you spoken with anyone else about how you've been feeling?.

3. Right Sidebar: The top contains a photo of a woman sitting in a chair in a clinical setting. Below the photo is a profile for Lucie Vitková, identifying her as a 25 year old female in the Psychiatry specialization. The bottom section, Available Tests, includes a search bar and a list of options: 1. Complete Blood Count, Blood pressure, Toxicology, and C T scan of the brain.

Navigate back to Image 1:.

### Image 2: Long description

The interface is divided into three main vertical sections.

1. Left Sidebar: Titled Conversations, it lists patient names including Lucie Vítková, Neurologický pacient 12, Klára Vránová, and others. Buttons at the bottom include Create Patient, New Evaluation, and Start Session.

2. Central Chat Area: Titled Virtual Patient BETA. It displays a dialogue history.

- A message bubble from 16:54 labeled Toxikologie contains a list of negative results for Ethanol, Paracetamol, Amphetamines, Cannabinoids, Cocaine, Opiates, Benzodiazepines, M D M A, Tramadol, Zolpidem, and Fentanyl.

- A second message bubble from 16:58 labeled C T mozku provides a text report of a computed tomography scan of the brain, stating normal symmetry, no signs of abnormalities, and normal brain anatomy.

- At the bottom is an input field with a prompt asking, Have you spoken with anyone else about how you've been feeling?

3. Right Panel:

- Top: A photo of a woman, Lucie Vítková, sitting in a chair in a clinical setting.

- Middle: Patient details listing Name as Lucie Vítková, Gender as Female, Age as 25 years old, and Specialization as Psychiatry.

- Bottom: Available Tests section with a search bar and buttons for Toxicology, C T scan of the brain, and C T scan of the body.

Navigate back to Image 2:.

### Image 3: Long description

The interface is divided into three main vertical sections.

1. Left Sidebar (Conversations): A list of patient names and session times, including Lucie Vítková, Neurologický pacient 12, Klára Vránová, and others. Buttons at the bottom allow for Create Patient, New Evaluation, and Start Session.

2. Central Panel (Chat History): A scrollable conversation log.

- Top messages show negative results for U-Zolpidem and U-Fentanyl.

- A file attachment icon labeled C T mozku (C T of the brain) is followed by a detailed medical report stating that the computed tomography scan shows normal brain anatomy and function with no signs of abnormalities.

- A diagnostic tag labeled Diagnosis: depression is followed by a long red-bordered text box titled Correct diagnosis. It identifies the condition as Adjustment Disorder (F 4 3 dot 2) with depressive syndrome, citing academic stressors and recommending psychotherapy and continued use of S N R I antidepressants.

- Bottom controls include Voice and Text input tabs and a large green Start Speaking button.

3. Right Sidebar (Patient Information and Tests):

- Top: A photo of a young woman sitting with her head down.

- Middle: Patient details for Lucie Vítková, Female, 25 years old, Specialization: Psychiatry.

- Bottom: Available Tests section with a search bar and options for Complete Blood Count and Blood pressure.

Navigate back to Image 3:.

